# Ingestion of caustic substances and its complications

**DOI:** 10.1590/S1516-31802001000100004

**Published:** 2001-01-02

**Authors:** Rui Celso Martins Mamede, Francisco Veríssimo de Mello

**Keywords:** Caustic esophagitis, Caustic substance ingestion, Inflammatory lesions of the upper digestive tract, Caustic stenosis, Esofagite cáustica, Ingestão de substâncias cáusticas, Lesões inflamatórias das vias digestivas superiores, Estenose cáustica

## Abstract

**CONTEXT:**

Caustic substances cause tissue destruction through liquefaction or coagulation reactions and the intensity of destruction depends on the type, concentration, time of contact and amount of the substance ingested.

**OBJECTIVES:**

To analyze the complications in patients who ingested caustic substances and correlate them with the amount of caustic soda ingested.

**DESIGN:**

Retrospective study.

**SETTING:**

University hospital, a referral center.

**PARTICIPANTS:**

A total of 239 patients who ingested caustic soda.

**MAIN MEASUREMENTS:**

The amount of granulated caustic substance ingested was measured as tablespoonfuls and the following complications were analyzed: esophagitis, esophageal stenosis and progression to cancer, fistulas, perforations, stomach lesions, brain abscesses, and death. Stenosis was classified as mild, moderate or severe according to the radiological findings.

**RESULTS:**

We observed an 89.3% incidence of esophagitis; 72.6% of the cases involved progression to stenosis and 1% died during the acute phase. Stenosis was mild in 17.6% of cases, moderate in 59.3% and severe in 23%. The incidence of stenosis was 80.8% in women and 62.5% in men. The incidence of stenosis was 46.9% in the group that ingested “fragments” and 93.6% in the group that ingested one or more tablespoonfuls of caustic substances. Among subjects who ingested one or more tablespoonfuls, 32.2% developed lesions of the stomach-duodenum, whereas the ingestion of “fragments” was not sufficient to induce these lesions. There was no correlation between the intensity of lesions of the esophagus and of the stomach. Progression to cancer of the esophagus occurred in 1.8% of cases, death during the chronic phase in 1.4%, perforations in 4.6%, fistulas in 0.9%, and brain abscesses in 1.4%.

**CONCLUSIONS:**

The complications were related to the amount of caustic soda ingested. Small amounts caused esophagitis or stenosis and large amounts increased the risk of fistulas, perforations and death.

## RESUMO

**CONTEXTO:**

As substâncias cáusticas promovem a destruição dos tecidos através de reação de liquefação ou coagulação, e a intensidade de destruição depende do tipo da concentração, do tempo de contato e da quantidade ingerida.

**OBJETIVOS:**

Analisar as complicações observadas e correlacioná-las com a quantidade de “soda cáustica” ingerida.

**TIPO DE ESTUDO:**

Estudo retrospectivo.

**LOCAL:**

Hospital Universitário, centro de referência.

**PARTICIPANTES:**

239 pacientes que ingeriram “soda cáustica”.

**VARIÁVEIS ESTUDADAS:**

Quantidade de cáustico granulado ingerido medido em colheres de sopa, e as complicações analisadas foram: esofagite, estenose e cancerização esofágica, fístulas, perfurações, lesões do estômago, abscesso cerebral e óbito. As estenoses foram classificadas em leve, moderada e grave, conforme os achados radiológicos.

**RESULTADOS:**

Observaram-se 89,3% de esofagite que em 72,6% evoluíram para estenose e 1% para óbito na fase aguda. Estenose leve foi encontrada em 17,6%, moderada em 59,3% e grave em 23%. Nas mulheres identificou-se 80,8% de estenose e no homem 62,5%. No grupo que ingeriu fragmentos houve 46,9% de estenose e no que bebeu uma ou mais colheres, 93,6%. Constatamos 32,2% de lesões no estômagoduodeno ao ingerirem uma ou mais colheres, pois a ingestão de “fragmentos” não foi suficiente para tal. Não houve, nesse estudo, correlação entre a intensidade de lesões do esôfago e do estômago. Observaram-se 1,8% de cancerização do esôfago, 1,4% de óbitos na fase crônica, 4,6% de perfurações, 0,9% de fístulas e 1,4% de abscesso cerebral.

**CONCLUSÕES:**

As complicações tiveram relação com a quantidade de soda cáustica ingerida; que pequenas quantidades causam esofagite ou estenose e, em grande quantidade, aumentam o risco de fístulas, perfurações e óbito.

## INTRODUCTION

The severity of lesions produced by caustic substances on tissue depends on the type, quantity and concentration of the caustic substance ingested, as well as on the time of contact with the mucosa.

Alkalis and acids have a great potential for tissue destruction. Due to their mechanisms of action, acids form scars on necrotic tissue, a fact that prevents their action at a greater depth. However, the inability of gastric juices to neutralize these substances contributes to the onset of lesions in various organs such as the stomach and intestine, in addition to the mouth and the esophagus.

On the other hand, the action mechanism for alkaline agents causes them to combine with tissue proteins to form proteinates, and with fats to form soap in addition to water. Thus, liquefaction necrosis occurs. The products formed favor the penetration of the remaining alkali into the tissue by increasing the solubility of the caustic soda, causing the alkalis to reach deeper tissue layers, consequently producing lesions in the entire thickness of the organ exposed to the substance. Alkalis promote blood thrombosis in blood vessels by base absorption, impairing irrigation of the esophagus.

However, alkali can be neutralized, at least in part, by gastric secretion, with a consequent reduction of its action on the mucosa of the stomach. For this reason, acids usually provoke the most severe lesions, especially in the antropyloric region. It should be remembered, however, that some individuals have achlorhydria, a situation in which the destruction produced by alkali is quite extensive. Similarly, since the esophagus has a slightly alkaline pH, its epithelium is resistant to acid, so that only 6 to 20% of those who ingest this substance present lesions in this organ.

With respect to the relationship between the severity of the lesion and the concentration of the substance ingested, it has been observed that the higher the concentration of the product, the greater its ability to provoke severe injury. In a study on rabbits,^1^ it was observed that the ingestion of a 3.8% solution of 1 N sodium hydroxide caused lesions of the mucosa, submucosa and internal plate of the esophageal musculature and that a 10.7% concentration of the same substance was sufficient to injure the muscle plate, while the ingestion of a 22.5% concentration fully destroyed the esophagus of the animals.

The effect of the time of contact of the caustic substance with tissue is similar to the effect of its concentration. In this respect, Holinger^2^ stated that one hour after accidental ingestion of a watch battery there were lesions of the mucosa; two to four hours later the lesion reached the muscle layer, and 8 to 12 hours later there was perforation of the esophagus. The effect of time of contact was also noted in different organs or even in different areas of the same organ. The esophagus, in contrast to the pharynx, is the organ presenting the most severe lesions, perhaps because the esophagus is of smaller caliber, with a consequent increase in time of contact.

Since in children the thymus compresses the esophagus, in the case of ingestion of caustic substances the tendency is towards more severe lesions in the upper third of the esophagus, as this compression delays the descent of the substance at this point. In contrast, in adults these lesions are more common in the area of compression provoked by the aortic-bronchial bifurcation and in the cardia area, which are more salient during this phase of life.

Other factors seem to contribute to the increase in time of contact between the caustic agent and the mucosa. Sugawa & Lucas^3^ stated that once the caustic agent is ingested, poor motility of the esophagus and the gastroesophageal reflux will increase the time of exposure, thus increasing the time of mucosal destruction. In this respect, a study on rabbits^4^ showed that gastroesophageal reflux retarded scarring of the lesion caused by the caustic agent.

A direct relationship is known to exist between the quantity of caustic substances ingested and the intensity of injury to the digestive tube. However, this conclusion was reached only in experimental studies, as it is difficult to determine the quantity of caustic substances ingested by patients, especially when a liquid product was ingested.

The objective of the present study was to investigate the complications observed in 239 patients who ingested caustic soda and who were admitted to the University Hospital of FMRPUSP, and to correlate them with the amount ingested.

## METHODS

The study was conducted on 261 patients who had ingested caustic substances and who were seen at the University Hospital of the Faculty of Medicine of Ribeirão Preto (a tertiary referral center) from 1957 to 1994. Of these, 239 had ingested caustic soda.

The medical records of the patients were surveyed in order to obtain information about the complications observed during the different phases of ingestion of caustic substances.

To determine the quantity of ingested caustic substance per patients, each patient was asked to compare the amount with an equivalent amount of crystal sugar measured in tablespoonfuls, considering that a tablespoonful contains 22 g of commercial caustic soda. This comparison was possible only because the patients had prepared their own “cocktail” using granulated caustic substances. When the patient reported that the ingested quantity was much less than 22 g, we defined this amount as “fragments” since it was impossible to determine exactly the amount ingested. Examples of these situations were children who had drunk water from a glass dirty with caustic soda, or children who had placed some flakes of caustic soda into their mouth. It was possible to obtain information about the quantity ingested for 70% of the patients (167/239).

We analyzed the possible complications according to different age ranges: 0-10 years, 11-20 years, 21-30 years, and more than 30 years and, when of interest, we compared them between 2 groups (children and adults).

To facilitate the process of description and the analysis of the degree of stenosis provoked in the esophagus by the caustic agent ingested, we prepared the classification described below. Our basis was a radiological study by means of a barium-swallow esophagogram performed immediately before the dilatations, i.e., two weeks after ingestion. To determine the sites of these lesions, we divided the esophagus into imaginary parts, i.e. upper, middle and lower thirds. The patients were divided into groups according to the following classification:

- *Absence of lesions:* when the esophagus showed no radiological lesions.
- *Mild stenosis:* when the esophagus presented one of the following images: an area of narrowing with irregular contours and loss of the mucosal pattern in one of the thirds; esophagus with slight rigidity and reduction in caliber, but with normal contour and mucosal relief.
- *Moderate stenosis:* moderate esophageal stenosis was considered to be present when one of the following images was obtained: esophagus tapered throughout its path, with reduced peristalsis and presenting delayed emptying; esophagus with narrowing in one of its thirds, with upstream dilatation and delayed emptying.
- *Severe stenosis:* when the esophagus showed: progressive tapering with more than 80% reduction of the lumen up to its distal portion, with irregular contours and loss of the mucosal pattern; filiform aspect throughout its extension, with or without signs of perforation, diverticula or fistulas.

The stomach and duodenum were studied in 93 patients by radiography, gastroscopy, or macroscopically during laparotomy.

### Statistical Methods

Data were processed and analyzed by descriptions of frequency and percentages and by statistical tests. To permit this, the 239 patients were considered to be a sample of the population. The data were fed into a PC 386 computer using the D Base software. Data analysis was performed using the EPI-5 software and, when necessary, the chi-squared test on a 0.001 basis. Fisher's exact test was used for data concerning less than 20 subjects.

## RESULTS

Among the 215 patients for whom there was full information, 192 progressed with complications related to esophageal lesions in 190 of them, and the last 2 died (0.8%) (Table 1). The 190 patients with some type of esophageal injury progressed in different manners according to the severity of the injury. A total of 156 cases (72.6%, 156/215) developed stenosis, whereas 34 (15.8%) healed with no effect on the caliber of the organ (esophagitis with superficial lesions). Most of these patients (58.8%, 20/34) were males, 67.6% (23/34) were patients who had ingested the caustic product accidentally, and 76.5% (26/34) were patients who drank caustic fragments. None of them had drunk more than three tablespoonfuls of caustic substance.

**Table 1:** Distribution by sex of complications during the acute phase of ingestion of caustic agents

Complications	Males		Females		Total	
	No.	%	No.	%	No.	%
Esophagitis	20	19.4	14	10.3	34	14.2
Stenosis	55	53.4	101	74.3	156	65.3
Death (acute)	01	1.0	01	0.7	02	0.8
Absent	13	12.6	10	7.3	23	9.6
Unknown	14	13.6	10	7.3	24	10.0
**Total**	**103**		**136**		**239**	

Stenosis occurred in 46.9% (23/49) of the patients who ingested “fragments” and in 93.6% (p>0.005) of the patients who ingested one or more tablespoonfuls of caustic.

In the present study, radiological examination permitted us to identify the sites of esophageal injury in 124 patients. Most of them had lesions in more than one esophageal third, with a 28.3% frequency of lesions involving the upper third, 31.9% involving the middle third and 29% involving the lower third. In 10.9% of cases, the entire esophagus was involved. Thus, the lesions were slightly more frequent in the middle esophageal third.

Stenosis of the esophagus was frequent in the different age ranges. The age group most frequently involved was from 11 to 20 years, with 94.9% of the patients developing stenosis, where as children were the least affected (49.2%), with a significant difference compared to the other age ranges (Table 2). Among adult patients, only 13.6% did not develop stenosis of the esophagus (Table 3).

**Table 2:** Distribution of the incidence of stenosis according to age range

Age Stenosis	Up to 10 years		11-20 years		21-30 years		>30 years	
	No.	%	No.	%	No.	%	No.	%
Absent	34	50.7	2	5.1	13	25.5	10	19.2
Present	33	49.2	37	94.9	38	74.5	42	80.8
**Total**	**67**		**39**		**51**		**52**	

**Table 3:** Frequency distribution of stenosis in children and adults. Stenosis was classified by radiological examination

Age Stenosis	Children		Adults		Total	
	No.	%	No.	%	No.	%
Mild	7	35.5	12	13.6	19	17.6
Moderate	11	55.0	53	60.2	64	59.3
Severe	2	10.0	23	26.1	25	23.1
**Total**	**20**		**88**		**108**	

Patients with stenosis of the esophagus concomitantly presented a 4.6% incidence (10/215) of perforations, a 0.9% incidence (2/215) of fistulas, a 1.4% incidence (3/215) of brain abscesses, a 1.4% death rate (3/215), and the occurrence of progression to cancer of the esophagus in 1.8% of the cases (4/215). These complications occurred in 63.3% of the patients who ingested two or three tablespoonfuls of caustic agents.

The stomach was examined in 93 patients by radiography or gastroscopy or during laparotomy, among which lesions were detected in 32.2% of the cases (30/93). Among these, 19.3% (18/93) were stenosis of the stomach, 2.1% (2/93) stenosis of the pylorus, 8.6% (8/ 93) stenosis of both structures, and 2.1% small mucosal lesions.

This type of injury occurred in 34.3% of the males (12/35) and in 31% of the females (18/58). We also observed that the ingestion of fragments was not sufficient to cause stomach injuries. The ingestion of one tablespoonful provoked lesions in 33.3% (9.27) of cases, the ingestion of two tablespoonfuls provoked lesions in 55.0% (11/20), and the ingestion of three tablespoonfuls caused lesions in 50.0% (4/8). Similarly, 51.7% (15/29) of the patients who vomited had lesions of the stomach and duodenum.

The distribution of caustic soda ingestion, as well as suicide attempts per decade, showed a major incidence (70 cases and 54 cases, respectively) in the 1980's (Tables 4 and 5). Incidence of injuries to the lower digestive tract was found to be 28.2% (13/46) up to 1985, and 34.5% (10/29) from 1986 to 1990, increasing to 50% (4/8) after 1990.

**Table 4:** Distribution of the amount of caustic soda ingested per decade

Time Quantity	1960-1969		1970-1979		1980-1989		1990-1994	
	No.	%	No.	%	No.	%	No.	%
Fragments	4	14.3	14	38.8	29	41.4	22	66.7
1 tablespoonful	11	39.2	11	30.5	23	32.8	2	6.0
2 tablespoonfuls	11	39.2	5	13.9	15	21.4	8	24.2
3 tablespoonfuls	2	7.1	6	16.7	3	3.0	1	3.0
**Total**	**28**		**36**		**70**		**33**	

**Table 5:** Frequency distribution of accidental caustic ingestion and suicide according to decade

Decade*	Accident		Suicide	
	No.	Rate	No.	Rate
60 (1,100,000 Pop.)	11	0.1	37	0.33
70 (1,375,000 Pop.)	21	0.15	28	0.20
80 (1,718,000 Pop.)	29	0.17	54	0.31
90 (2,150,000 Pop.)	20	0.18	18	0.16
**Total**	**81**		**137**	

* *The population (Pop.) value corresponds to the population of Ribeirão Preto and region during the decades studied.*

Of the 239 patients who drank caustic soda, 4 of them (1.6%) developed cancer of the esophagus. One was a male and three were females, with a mean age of 51 years. Among the four patients who acquired cancer of the esophagus, two ingested the caustic soda accidentally and the others while attempting suicide; these patients ingested amounts ranging from fragments to as much as three tablespoonfuls. Two were classified as having moderate stenosis and two as having severe stenosis. Stenosis was localized in the middle or lower third or in the entire organ and was dilated with Hurst, Plummer, Jackson and Tucker tubes and for this reason the patients kept a gastrostomy for periods of between 3 and more than 10 years.

It can be seen that the correlation between stenosis of the esophagus and injuries to the stomach and duodenum is not always present since two patients whose esophagus was preserved had lower lesions and, conversely, of 17 patients with severe esophageal injuries, 9, i.e. 52.9% (9/17), had no stomach lesions. Only one patient with injuries to both the stomach and duodenum had injuries in the middle and upper thirds of the esophagus, while the remaining ones had stenosis in the middle and lower third or throughout the extension of the organ (Table 6).

**Table 6:** Distribution of lesions of the esophagus, stomach and duodenum by degree of severity

Stomach-duodenum	Esophagus				
	Preserved	Esophagitis	Mild stenosis	Moderate stenosis	Severe stenosis
preserved	11	-	7	25	9
mild lesions	1	-	-	-	1
stenosis	1	3	2	9	7
**Total**	**13**	**3**	**9**	**34**	**17**

## DISCUSSION

The physiopathological characteristics of the caustic lesions undergo transformation over the following four phases:

During the first hours there is eosinophilic necrosis with edema and intense hemorrhagic congestion (caustic esophagitis).

During the first days the ulcerations are covered with a leukocytic fibrinous layer. Perforation will occur if ulceration exceeds the muscle plane. Fibroblasts reach the site after about 4 days. On the 5^th^ day, a mold of the lesion is formed and when the lesion is extensive, covering the entire esophagus, the esophageal mold is formed. The mold consists of dead cells, secretions and food remains.

Repair occurs during the first weeks, especially after the 10^th^ day. Edema will persist in the submucosa, together with lymphatic ectasia. Sclerosis sets in at the muscle level and the autonomic nervous plexus is destroyed. Fibrosis occurs in layers whose depth depends on the severity of the caustic injury (caustic stenosis).

During the first month, epithelialization of the mucosal ulcerations occurs with difficulty due to the vascular lesions.

For the rest of their lives, these patients may present new ulcerations followed by re-epithelialization due to small traumas provoked by food. These traumas increase the scars, reducing even more the lumen of the organ. These are the mechanisms of late stenosis and of the recurrence of previously dilated stenoses.

Only studies carried out in Turkey^5^ and Denmark^6^ have identified such high levels of injury as observed here, i.e. incidence of esophageal stenosis of 72.7% and 85%, respectively, among patients who ingested caustic agents. In contrast, the incidence reported in Finland^7^ was extremely low, since the cited authors stated that the commerce of caustic soda was prohibited in Finland in 1966. These investigators reported 98 cases of children who ingested soap and vinegar, with only 20 of them suffering esophageal injury, but with no stenosis of the organ in any case.

Some investigators have discussed the influence of the physical status of the caustic agent ingested on the severity of the lesions produced in the human esophagus. In our study the patients had ingested caustic soda exclusively in the solid form, diluted in half a glass or a full glass of water or strong spirits. Shikowitz et al.^8^ stated that soda in the solid form is less aggressive to the esophagus and justified this fact by stating that the product sticks to the oral mucosa, where it produces deep lesions. When comparing solid and liquid soda, Kikendall^9^ stated that the liquid form at concentrations of less than 10% only causes esophageal stenosis as in the solid form, whereas the concentrated liquid form can provoke more lesions in the stomach than the solid form, as well as producing more severe perforations and stenosis in the esophagus. This was not what we observed in our patients, 73.5% of whom (158/215) had severe lesions of the esophagus or died when they ingested caustic soda in the solid form.

In a study of 202 children, Gundogdu et al.^5^ observed that the upper third of the esophagus was involved in 40.6% of cases, followed by the middle third in 23.8%, by the lower third in 23.3%, and by injury to the entire esophagus in 12.4% of cases. The incidence of lesions of the upper esophageal third was lower in the present study than in the one cited above. We believe that this was due to the fact that our sample contained a smaller number of children than of adults. It is known that, in caustic ingestion, children tend to have stenosis of the upper third, whereas adults have stenosis of the middle and lower thirds. Naturally the healing process will depend on the extent of injury provoked by the caustic substance. If it is simply lesions of the epithelial layer, a cure will occur rapidly even without treatment. When the burn reaches the submucosal tissue, cicatricial reins, half-moon stenosis or membranous narrowing will appear. Near the stenosis zones, the muscle layer hypertrophies and suffers spasms, impairing the dilating treatment. The most intense lesions reach the periesophageal tissue, where they provoke adhesions that impair the closing of the cardia or even cause shortening of the organ. Pavliuk^10^ stated that the stenosis occurring in the middle third is due to failure of the microcirculation at this level, which is therefore the preferred site for the installation of stenosis.

Analysis of the patient group that developed stenosis showed a significantly higher number of women with this complication, i.e. 80.8% (101/125), as opposed to 62.5% (55/88) of the men. This fact leads us to infer that females may be more sensitive to caustic agents or that they ingested a larger amount of caustic substance than males. If we consider only the patients of both sexes who drank caustic fragments and who developed stenosis, we can see that there was no significant difference in the frequency of stenosis between males (31.4%) and females (41.4%). This suggests that the statistically significant difference detected between sexes may have been related to the amounts of caustic agent ingested by men and women. This observation makes sense, since analysis of the amount of caustic substance ingested by each sex shows that men ingested more fragments than women did.

Stenosis occurred in 46.9% (23/49) of the patients who ingested “fragments” and in 93.6% of the patients who ingested one or more tablespoonfuls of caustic. Once again it is clear that the presence of stenosis of the esophagus is directly related to the amount of caustic agent ingested and that when the amount ingested is close to one tablespoonful the esophagus will be sufficiently injured. With the ingestion of two or three tablespoonfuls, the risk of fistulas, perforations or even death is increased. This leads us to believe that the patient who ingests more than 60 grams, i.e. three tablespoonfuls of caustic substance, will probably die. It has been reported^11^ that 50 cc of concentrated liquid is, on average, sufficient to provoke extremely severe injuries, while an amount of 15-30 cc causes severe lesions, and less than 15 cc causes lesions of medium intensity. On this basis, we were surprised with the high incidence of stenosis among patients who had only ingested caustic fragments in our samples, since we expected a lower incidence.

Young subjects aged 11 to 20 years were expected to have higher indices of stenosis and younger children were expected to have the lowest indices, as adolescents ingest caustic agents with the intent of suicide, while children do so accidentally and, as is well known, a smaller amount of caustic agent is ingested accidentally. Children may be exposed to caustic substances as concentrated as those to which adults are exposed, such as products used in rural areas, but when they ingest these products their lesions are less intense because pain limits the amount swallowed.^9^

However, it has also been reported that, regardless of the treatment instituted, 7-15% of the children who have contact with caustic substances develop stenosis of the esophagus.^12^

In our sample, we observed higher rates than reported by the cited authors (49.2%), perhaps because we opted to study only patients who ingested caustic soda, which is known to be the most aggressive caustic substance. In agreement with this idea, our data show that even though 93.7% of the children ingested small quantities of caustic agent, i.e. “fragments”, 50% of them presented stenosis. When the stenosis was classified by radiology, the children were found to show a 10% rate of severe stenosis (Table 4). Thus, the fact they ingested caustic fragments does not eliminate the possibility of stenosis, even of a severe grade. We believe that the severity of esophageal lesions depends, in addition to the time of contact of the substance with the organism, on the amount ingested and on its concentration. These data lead us to believe that when the patient places into his mouth a caustic agent in the solid state in the form of flakes, few flakes are swallowed, although the ones that are swallowed are highly concentrated since they are diluted only by the saliva present in the mouth. It has been previously demonstrated^13^ that lesions provoked by caustic soda are the most severe, and are more serious than those provoked by alkaline agents present in dishwashing products.

The aggressiveness of caustics seems to be lower among adult patients since, if we consider only ingestion of caustic fragments, the incidence of stenosis was lower among adults (16.7%) than among children (40.4%), although the association tests did not show a significant difference. No difference in the incidence of stenosis was observed between the various age ranges of children, i.e. the frequency of stenosis among children seems to be independent of age.

Analysis of the occurrence of stenosis as a complication of caustic ingestion over the historical series studied showed that the frequency of stenosis has been decreasing among patients who ingested caustic substances over the last few years. In 1969 the incidence of stenosis was 98.4%, as opposed to a current incidence of 37.9%. A significant decrease in the incidence of esophageal burns was observed in Denmark in a study on 102 children,^14^ and the same fact was observed in the USA^15,16^ and Russia.^17^ The cited investigators attribute this decrease to changes in the composition of home products and to the introduction of containers that are difficult for children to handle. Since in Brazil there is no specific law concerning reduction of caustic concentrations in commercial products, the hypothesis of a reduction of stenosis due to lower caustic concentration in home products is unlikely. Although no changes in caustic concentrations occurred, it can be seen that patients are ingesting progressively smaller amounts, as the incidence of suicide is decreasing.

According to Shikowitz et al,^8^ many variables affect the incidence of stomach injuries, such as tonus of the pylorus and type of food ingested before the caustic agent. These data were not surveyed in the present study because of the difficulty in obtaining information in a retrospective study.

The presence of a 32.2% rate of stomach and duodenal injuries observed in the present study, which exclusively considered the ingestion of caustic soda in the solid form, is in contrast to studies by other investigators^8,9^ who believed that this type of caustic agent hardly reaches the stomach because, according to them, it is retained along the trajectory. We think that the administration of an antidote, milk and fluids to some of our patients may have contributed to the presence of injuries in the stomach and duodenum.

We believe that the increase in stomach and duodenum injuries could be explained by the introduction of H2 blockers in the therapy indicated at our service. It is possible that these blockers, administered as soon as the patient arrives in order to neutralize reflux, are permitting deeper lesions of the stomach, probably by neutralizing the acid of gastric juice. For this reason, we propose that this treatment should be administered 24 hours after caustic ingestion.

Drinking an antidote, passing a nasogastric tube, and corticoid or antibiotic treatment showed no effect on the incidence of these complications. The occurrence of brain abscesses during the acute phase has been considered to be probably due to brain metastases of esophageal infection.^18^

Several investigators attribute significant importance to the presence of vomiting as a factor contributing to the aggressiveness of injury.^2,19^ This factor was observed in our sample when we studied the influence of vomiting on caustic aggression against the mucosa, since a 6.9% incidence (4/58) of complications was observed among patients who vomited during the acute phase, whereas no complications were observed among those who did not vomit.

Five of the 239 patients studied (2.0%) died, two of them during the acute phase of caustic ingestion and three during the chronic phase. One of the patients died a few hours after admission to the hospital due to organ necrosis. The other died on the 12^th^ day due to hemorrhage caused by the rupture of large vessels. The causes of these deaths agreed with those reported in the literature. According to Christesen,^6^ acid leads to death by causing gastric perforation, stenosis of the pylorus and massive metabolic acidosis, while the alkali provokes esophageal stenosis, tracheal necrosis or even perforation of the heart, as reported by Ranzato et al.^20^

Among the patients who died during the chronic phase, one died of complications of an esophagotracheal fistula acquired when he ingested the caustic substance and the others died as a consequence of perforations of the esophagus provoked by dilatation. Four ingested the caustic agent while attempting suicide. The child who died underwent dilatation for more than 10 years and died of a perforated esophagus during dilatation.

Another complication of caustic ingestion observed in our patients during the chronic phase was progression to cancer of the esophagus burned by the caustic agent and submitted to frequent traumas induced by dilating tubes and food stasis. The mean age of these patients with cancer of the esophagus caused by caustic ingestion (50 years) was significantly lower than the age of patients with cancer of the esophagus due to other etiologies and their prognosis was usually better. The cancers developed in the middle and lower thirds, except for one case in the upper third. This is in contrast to a report by Titk^21^ who stated that this cancer is of the spinous cell type and that the lesion tends to localize at the level of the bronchial bifurcation or below it.

Most patients who developed cancer were young when they ingested the caustic agent: one was 17 years old (ingested in 1961), one was 20 (ingested in 1942) and one was 22 and ingested in 1967. The 7-year-old child ingested caustic in 1946. These patients had ingested caustic substance on average 30 years (20-46) earlier and had undergone to dilatation of the esophagus for part of this time. According to Andreoni et al,^22^ the tumor always appears 15 to 40 years after caustic ingestion. Thirty-seven of our patients who had ingested a caustic agent more than 15 years before were being followed up. Considering this risk population, the rate of cancer development was 10.8%. Also, according to Andreoni et al,^22^ the possibility of the occurrence of cancer of the esophagus among burn patients is 3000 times higher than among the population at large. For this reason, esophagectomy has been indicated as a form of preventing cancer in an esophagus burned by caustic agents.^3,23^ Eaton and Tenneekoon reported that the incidence of cancer of the stomach after caustic ingestion is not excessive.^24^

Current popular belief is that caustic substances do not kill, which is not true, but when death does not occur, stenosis of the esophagus will inevitably develop causing the patients to depend on dilatation, with the risk of perforation or progression to cancer.

We believe that guidance and education are important preventive tools, but the best weapon is to restrict access to caustic agents, especially caustic soda, recognized as the most potent, by prohibiting their free commercialization.

## CONCLUSION

On the basis of the present data concerning the ingestion of caustic soda, we conclude that: a) the complications were related to the amount of caustic agent ingested, with stenosis of esophagus, of stomach and deaths; b) there was esophagitis and stenosis, and the ingestion of large amounts of caustic agent increased the risk of fistulas, perforations and deaths.
